# Experimental dataset from a central composite design to develop mortars with self-compacting properties and high early age strength

**DOI:** 10.1016/j.dib.2021.107563

**Published:** 2021-11-12

**Authors:** Lino Maia

**Affiliations:** a CONSTRUCT-LABEST, Faculty of Engineering (FEUP), University of Porto, Rua Dr. Roberto Frias, Porto 4200-465, Portugal; b Faculty of Exact Sciences and Engineering, University of Madeira, Campus da Penteada, Funchal 9020-105, Portugal

**Keywords:** Cement, Commercial sand, Design of experiments, Mortar, Response model

## Abstract

The concrete workability and the compressive strength are the principal properties of the fresh and hardened concrete, respectively. When self-compacting properties are required, scientific knowledge is important and appropriate models applied to achieve optimized compositions. Here, experimental data regarding to the mortars is presented. The dataset regards to a design of experiments carried out in mortars with commercial materials through a central composite design with five independent variables: Water_v_/Cement_v_, Superplasticyzer_m_/Powder_v_, Water_v_/Powder_v_, Sand_v_/Mortar_v_, FineSand_v_/Sand_v_. In total 64 mortar composition were done: 2^5^ factorial design consisting on 32 treatment combinations augmented by 10 axial runs plus 8 central runs, resulting in a central composite design with 50 mortar trial mix composition. Beside 14 extra mixes were done to allow comparing and validating results for the response models to be applied. Four dependent variables were measured: the D-flow and the t-funnel to measure the workability and the tensile strength and the compressive at the age of 24 h to assess the mechanical properties. Since the experiments were run based in a central composite design and extra mixes were prepared, response models can be applied to the dataset in order to find optimized mix compositions.

**Specifications Table**

**Table:** 

Subject	Civil and Structural Engineering.
Specific subject area	Construction materials, concrete technology, self-compacting concrete, mortars with self-compacting properties.
Type of data	Tables.
How the data were acquired	Data was acquired through readings in laboratory tests. The design of experiments (mortar mix compositions) was defined based on a central composite design. Then, after mixing the mortar workability was assessed by the flow and the V-funnel tests and the early age mechanical properties were assessed by the tensile strength and the compressive strength at the age of 24 h.
Data format	Raw.
Description of data collection	Just after mixing the time in the V-funnel was done and immediately after the slump test was done. Just after these tests, three prismatic specimens 4 × 4 × 16 [cm] were moulded and stored in a climatic chamber at 20 °C. At the age 23.5 h the specimens were unmoulded and mechanically tested at the age of 24 h (with 15 min tolerance). First the tensile strength test was done, and two halves were obtained by specimen. Immediately after that, the compressive strength was done for the six halves.
Data source location	• Institution: Faculty of Civil Engineering of the University of Porto • City: Porto • Country: Portugal
Data accessibility	With the article

**Value of the Data**

• Most of data published in papers is from experiments carried out only with for three independent variable and with the normalized sand; that means, typically no more than 20 experiments. Here I used two commercial sands, which means an extra independent variable. Beside, the total volume of sand was an independent variable, too. With five independent variables the central composite design changed done 50 mix compositions to be tested. Additionally, here I have 14 compositions available for validations.
• With 64 mix composition testes (50 in a Central Composite Design), the next coming models, outliers/errors analysis and statistical analysis are better supported and surpassed by the number of experiments.
• Since two commercial sands were used with the content ratio being tested, the expecting next findings are much more connected to the reality than in experiments with normalized sand. Beside, the effect of the volume of sand may also be analyzed.
• Researchers that deal with self-compacting properties and/or high early age strength in mortars, especially the ones that are working/developing or applying or validating models may be interested in this data. Similarly, researchers that are dealing the statistics and approaches in concrete or mortars and/or, that are looking for data to apply in designing mortars and concrete with artificial intelligence may be interested in this data.
• This data may be applied to develop response models, may be used as data for artificial intelligence and/or may be used for statistics purposes.

## 1 Data Description

Table 1 presents the correspondence between the levels and the real values.

**Table 1:** Correspondence between the coded and real values.

Level	−α	−1	0	1	α
Water_v_/Cement_v_	0.68197	0.80531	0.89478	0.98426	1.10760
Water_v_/Powder_v_	0.019116	0.021719	0.023608	0.025496	0.028100
Water_v_/Powder_v_	0.43443	0.51300	0.57000	0.62700	0.70557
Sand_v_/Mortar_v_	0.36584	0.43200	0.48000	0.52800	0.59416
FineSand_v_/Sand_v_	0.04324	0.25000	0.40000	0.55000	0.75676

Table 2 presents the dataset for the mix compositions of the mortars prepared for 1.529 L based on a complete central composite design for five independent variables. Beside, it includes 14 extra mix compositions done for validation of response models purposes. The Table 2 includes the coded values, and the mix constituent contents. Note that, in Table 2 the total water added to the mixes is the result of the water effective plus the water for the absorption of the aggregates minus the water included in the superplasticiser content.

**Table 2:** Mix compositions produced.

Standard Order	Run Order	Water_v_/ Cement_v_	Water_v_/ Powder_v_	Water_v_/ Powder_v_	Sand_v_/ Mortar_v_	FineSand_v_/ Sand_v_	Cement [g]	Filler [g]	Superplasticiser [g]	Fine Sand[g]	Medium Sand [g]	Water Added [g]
1	47	−1	−1	−1	−1	−1	1133.5	558.3	12.466	434.3	1297.9	293.03
2	42	1	−1	−1	−1	−1	927.4	736.5	12.466	434.3	1297.9	293.03
3	30	−1	1	−1	−1	−1	1133.5	558.3	14.634	434.3	1297.9	291.73
4	14	1	1	−1	−1	−1	927.4	736.5	14.634	434.3	1297.9	291.73
5	2	−1	−1	1	−1	−1	1288.3	316.7	11.593	434.3	1297.9	333.77
6	23	1	−1	1	−1	−1	1054.0	519.2	11.593	434.3	1297.9	333.77
7	12	−1	1	1	−1	−1	1288.3	316.7	13.609	434.3	1297.9	332.56
8	19	1	1	1	−1	−1	1054.0	519.2	13.609	434.3	1297.9	332.56
9	49	−1	−1	−1	1	−1	941.9	464.0	10.359	530.8	1586.3	245.87
10	7	1	−1	−1	1	−1	770.6	612.0	10.359	530.8	1586.3	245.87
11	25	−1	1	−1	1	−1	941.9	464.0	12.161	530.8	1586.3	244.79
12	26	1	1	−1	1	−1	770.6	612.0	12.161	530.8	1586.3	244.79
13	33	−1	−1	1	1	−1	1070.5	263.2	9.633	530.8	1586.3	279.73
14	10	1	−1	1	1	−1	875.9	431.5	9.633	530.8	1586.3	279.73
15	24	−1	1	1	1	−1	1070.5	263.2	11.309	530.8	1586.3	278.72
16	32	1	1	1	1	−1	875.9	431.5	11.309	530.8	1586.3	278.72
17	5	−1	−1	−1	−1	1	1133.5	558.3	12.466	955.4	778.7	291.99
18	40	1	−1	−1	−1	1	927.4	736.5	12.466	955.4	778.7	291.99
19	28	−1	1	−1	−1	1	1133.5	558.3	14.634	955.4	778.7	290.69
20	41	1	1	−1	−1	1	927.4	736.5	14.634	955.4	778.7	290.69
21	3	−1	−1	1	−1	1	1288.3	316.7	11.593	955.4	778.7	332.73
22	34	1	−1	1	−1	1	1054.0	519.2	11.593	955.4	778.7	332.73
23	31	−1	1	1	−1	1	1288.3	316.7	13.609	955.4	778.7	331.52
24	20	1	1	1	−1	1	1054.0	519.2	13.609	955.4	778.7	331.52
25	4	−1	−1	−1	1	1	941.9	464.0	10.359	1167.7	951.8	244.61
26	18	1	−1	−1	1	1	770.6	612.0	10.359	1167.7	951.8	244.61
27	9	−1	1	−1	1	1	941.9	464.0	12.161	1167.7	951.8	243.53
28	37	1	1	−1	1	1	770.6	612.0	12.161	1167.7	951.8	243.53
29	11	−1	−1	1	1	1	1070.5	263.2	9.633	1167.7	951.8	278.46
30	6	1	−1	1	1	1	875.9	431.5	9.633	1167.7	951.8	278.46
31	38	−1	1	1	1	1	1070.5	263.2	11.309	1167.7	951.8	277.46
32	21	1	1	1	1	1	875.9	431.5	11.309	1167.7	951.8	277.46
33	45	−α	0	0	0	0	1312.1	222.8	11.955	772.0	1153.6	287.63
34	46	α	0	0	0	0	807.9	658.7	11.955	772.0	1153.6	287.63
35	27	0	−α	0	0	0	1000.0	492.6	9.680	772.0	1153.6	288.99
36	35	0	α	0	0	0	1000.0	492.6	14.229	772.0	1153.6	286.26
37	13	0	0	−α	0	0	834.2	764.2	13.085	772.0	1153.6	239.09
38	16	0	0	α	0	0	1139.5	264.2	11.004	772.0	1153.6	328.45
39	44	0	0	0	−α	0	1219.5	600.7	14.579	588.4	879.3	347.96
40	48	0	0	0	α	0	780.5	384.5	9.330	955.7	1428.0	227.30
41	17	0	0	0	0	−α	1000.0	492.6	11.955	83.5	1839.6	288.99
42	39	0	0	0	0	α	1000.0	492.6	11.955	1460.6	467.7	286.26
43	15	0	0	0	0	0	1000.0	492.6	11.955	772.0	1153.6	287.63
44	1	0	0	0	0	0	1000.0	492.6	11.955	772.0	1153.6	287.63
45	29	0	0	0	0	0	1000.0	492.6	11.955	772.0	1153.6	287.63
46	36	0	0	0	0	0	1000.0	492.6	11.955	772.0	1153.6	287.63
47	22	0	0	0	0	0	1000.0	492.6	11.955	772.0	1153.6	287.63
48	43	0	0	0	0	0	1000.0	492.6	11.955	772.0	1153.6	287.63
49	8	0	0	0	0	0	1000.0	492.6	11.955	772.0	1153.6	287.63
50	50	0	0	0	0	0	1000.0	492.6	11.955	772.0	1153.6	287.63
	51	0.63	3.05	0.21	0.52	−2.00	907.0	498.2	14.052	203.1	1820.6	277.58
	52	0.63	1.50	0.21	0.52	−2.00	907.0	498.2	12.647	203.1	1820.6	278.42
	53	0.63	1.50	0.21	0.52	−2.67	907.0	498.2	12.647	0.0	2022.9	278.82
	54	−0.13	0.21	−0.66	−0.62	0.00	1025.3	584.0	13.176	723.8	1081.5	290.09
	55	1.08	−0.42	−1.13	−0.62	0.00	882.7	733.5	12.739	723.8	1081.5	280.59
	56	−1.34	0.52	−0.14	−0.62	0.00	1210.5	396.1	13.232	723.8	1081.5	300.45
	57	−0.82	0.58	−0.39	−0.62	0.00	1123.2	484.9	13.418	723.8	1081.5	295.37
	58	0.57	−0.15	−0.92	−0.62	0.00	940.0	672.7	12.928	723.8	1081.5	284.70
	59	−0.13	−0.42	−0.66	−0.62	0.00	1025.3	584.0	12.517	723.8	1081.5	290.48
	60	−0.13	−0.42	−0.66	−0.62	0.00	1025.3	584.0	12.517	723.8	1081.5	290.48
	61	1.08	−0.42	−1.13	−0.62	0.00	882.7	733.5	12.739	723.8	1081.5	280.59
	62	−1.34	0.52	−0.14	−0.62	0.00	1210.5	396.1	13.232	723.8	1081.5	300.45
	63	−0.82	0.58	−0.39	−0.62	0.00	1123.2	484.9	13.418	723.8	1081.5	295.37
	64	0.57	−0.15	−0.92	−0.62	0.00	940.0	672.7	12.928	723.8	1081.5	284.70

Table 3 presents the dataset for the raw reading values obtained in the tests. The Table 3 regards to: V-funnel teste (t-funnel), flow teste (D-flow1, D-flow2), tensile strength teste (TS1, TS2, TS3), compressive strength teste (fc1.1, fc1.2, fc2.1, fc2.2, fc3.1, fc3.2).

**Table 3:** Values obtained in the tests.

Standard Order	Run Order	t-funnel [s]	D-flow1 [mm]	D-flow2 [mm]	TS1 [MPa]	TS2 [MPa]	TS3 [MPa]	fc1.1 [MPa]	fc1.2 [MPa]	fc2.1 [MPa]	fc2.2 [MPa]	fc3.1 [MPa]	fc3.2 [MPa]
1	47	18.89	33.00	33.00	12.13	12.36	10.32	73.98	74.26	70.75	70.76	72.18	73.30
2	42	12.26	34.90	34.90	11.34	11.30	10.50	56.50	56.38	58.07	55.75	57.99	62.09
3	30	16.70	33.70	34.00	12.90	13.40	11.63	73.42	72.29	67.02	69.33	67.76	70.74
4	14	11.32	35.60	35.30	11.02	10.84	9.65	56.75	55.01	55.05	55.42	56.56	57.54
5	2	9.24	38.10	36.90	11.01	11.68	11.08	68.92	67.27	68.03	69.41	64.68	63.96
6	23	7.08	37.00	36.90	9.43	9.96	8.90	57.61	53.71	54.63	54.01	58.53	55.83
7	12	7.80	37.30	37.90	10.23	11.52	10.05	69.71	69.45	68.35	65.48	69.02	68.82
8	19	6.91	38.00	37.00	10.48	9.99	10.07	54.94	53.47	51.88	52.33	53.81	53.95
9	49	114.06	25.20	25.40	10.88	12.12	12.05	75.01	71.51	73.75	73.32	71.10	74.07
10	7	38.91	29.10	28.80	10.75	12.08	12.09	57.46	58.20	54.96	61.46	58.93	59.90
11	25	74.32	26.00	26.10	12.30	9.12	10.03	73.17	70.48	72.15	72.01	71.58	71.47
12	26	33.03	28.60	29.30	10.20	11.37	11.17	60.71	58.57	57.32	59.85	60.39	58.96
13	33	25.40	29.40	29.60	11.35	12.36	11.78	65.94	67.17	66.40	70.08	69.59	67.62
14	10	17.53	31.00	31.70	11.48	11.34	n.av.	53.96	56.24	55.25	57.30	56.17	56.67
15	24	26.33	30.10	30.60	11.84	12.01	10.38	71.98	71.70	68.19	65.43	67.01	66.28
16	32	14.15	32.30	31.70	10.74	10.87	11.17	56.10	56.50	56.03	56.55	57.18	56.53
17	5	28.43	30.20	29.80	11.06	11.14	10.99	68.20	66.00	64.15	68.31	66.02	66.55
18	40	12.89	35.00	35.30	11.51	11.39	9.18	56.04	56.75	52.95	57.43	56.88	55.52
19	28	17.67	33.20	33.70	10.33	13.03	11.34	70.57	71.68	66.55	68.87	69.08	70.81
20	41	11.73	34.80	35.10	11.48	10.06	10.65	54.46	53.12	55.02	56.57	56.00	55.44
21	3	9.90	38.20	37.40	11.09	10.86	11.66	66.66	62.54	66.95	67.10	66.43	65.82
22	34	7.16	38.00	37.90	9.51	10.36	9.92	54.88	53.68	54.38	54.86	54.70	54.60
23	31	8.54	38.00	39.10	10.05	11.29	10.79	66.51	65.92	62.22	63.21	64.21	64.25
24	20	6.94	38.10	38.30	10.09	10.43	10.26	53.76	53.53	53.79	54.91	52.21	n.av.
25	4	*	23.50	23.10	12.01	11.93	12.36	68.28	66.93	65.55	68.73	68.11	66.43
26	18	52.64	27.70	27.30	10.65	11.55	12.07	54.95	55.47	54.85	54.48	56.96	55.11
27	9	67.34	26.70	26.40	12.73	13.35	12.29	69.70	68.83	68.74	66.74	67.00	70.24
28	37	33.30	28.40	28.60	9.52	11.83	10.09	57.04	57.20	54.89	55.62	55.66	55.70
29	11	26.20	29.10	28.80	12.22	13.03	10.69	66.52	64.62	66.38	66.83	63.45	62.93
30	6	16.51	31.60	31.40	11.50	11.06	10.38	51.63	52.92	52.51	52.09	56.12	55.07
31	38	18.57	31.10	30.70	12.53	12.15	10.95	61.64	59.09	58.92	59.53	64.67	61.91
32	21	17.07	31.20	31.60	9.50	11.94	10.55	53.47	53.29	53.31	55.65	52.88	51.85
33	45	*	16.90	16.70	12.56	11.26	11.92	79.07	78.09	78.93	77.13	80.34	76.96
34	46	11.55	34.10	34.30	9.13	10.74	10.87	50.02	47.91	48.72	49.16	48.05	49.68
35	27	17.64	33.20	32.90	12.11	11.17	11.06	62.38	60.61	61.92	63.09	65.15	61.51
36	35	12.71	33.80	33.60	10.97	11.47	n.av.	60.01	57.19	58.39	57.69	59.94	60.13
37	13	79.63	29.50	29.50	12.44	11.88	12.00	64.62	63.10	64.33	62.21	65.48	65.05
38	16	7.27	36.80	36.90	10.71	9.70	10.12	62.53	64.17	63.18	62.54	60.06	65.75
39	44	7.49	39.90	39.70	n.av.	9.51	9.35	64.84	59.79	58.15	60.31	56.78	58.74
40	48	*	16.60	17.30	10.37	9.87	10.73	63.23	63.40	61.52	60.49	63.17	63.93
41	17	16.55	33.50	34.10	n.av.	n.av.	n.av.	61.64	60.28	64.54	63.37	61.73	61.69
42	39	16.14	33.00	33.10	n.av.	n.av.	n.av.	58.81	56.35	57.82	56.17	59.32	58.00
43	15	14.98	34.70	34.60	11.84	12.82	10.66	60.59	58.04	58.83	59.72	61.73	60.45
44	1	13.77	34.60	34.60	11.67	12.66	12.55	61.09	62.64	63.86	63.85	60.85	60.30
45	29	14.15	33.60	34.30	10.17	11.53	12.15	59.47	60.73	60.41	57.16	59.42	58.64
46	36	14.74	34.80	34.80	11.92	12.20	12.13	60.14	59.88	60.37	63.04	59.91	62.08
47	22	14.53	34.10	33.80	12.15	12.87	10.87	61.53	58.30	60.23	59.81	60.17	59.97
48	43	14.38	34.10	34.10	10.92	12.52	12.22	60.65	62.77	62.02	61.20	65.90	60.45
49	8	13.56	34.40	34.60	10.56	11.04	11.54	61.24	61.10	58.69	59.84	59.36	60.85
50	50	14.23	34.40	34.50	10.26	11.73	11.11	59.55	59.28	59.81	62.26	58.62	58.24
	51	16.17	32.00	32.10	11.66	11.77	11.68	59.26	58.73	57.34	58.34	58.75	58.27
	52	16.58	32.70	32.70	9.16	11.82	12.30	61.79	61.47	60.15	59.10	59.64	61.01
	53	18.33	31.00	31.10	11.28	11.02	11.20	62.95	60.83	60.95	61.94	60.35	62.21
	54	12.96	35.20	35.40	11.72	12.78	11.56	63.04	58.89	62.64	61.85	60.12	61.42
	55	14.09	33.70	33.80	10.72	12.38	11.31	55.46	56.76	57.69	57.89	55.97	56.43
	56	14.15	34.60	34.80	11.77	9.30	9.99	72.67	71.09	69.75	71.96	71.16	66.91
	57	13.64	34.10	33.60	10.03	9.27	9.75	63.48	66.92	66.39	69.04	66.72	63.19
	58	13.02	33.50	33.50	9.58	11.68	12.23	61.89	58.93	59.85	58.42	57.76	57.83
	59	14.76	35.00	35.00	14.64	12.18	13.05	61.67	63.98	63.00	65.40	n.av.	n.av.
	60	15.01	34.30	34.60	11.78	10.68	10.97	61.97	62.12	72.45	65.15	64.59	64.68
	61	14.32	34.50	34.80	10.98	12.43	11.87	59.46	57.66	56.87	56.15	57.86	55.48
	62	15.65	34.40	34.50	13.90	13.95	14.05	73.59	69.67	77.18	72.45	73.03	69.83
	63	15.26	34.40	34.70	13.92	12.84	12.59	70.20	66.49	67.56	69.09	69.97	70.45
	64	15.08	34.80	34.70	n.av.	n.av.	n.av.	57.13	58.41	59.04	60.83	60.29	61.56

n.av.: The reading value is not available due to problems in the press equipment.

⁎ The mortar was too dry and blocked the V-funnel.

## 2 Experimental Design, Materials and Methods

Commercial materials available in the Portuguese market were used. In this experimental program two powders and two sands were used. The powders were the cement (ρ = 3.1 g/cm^3^) CEM I 42.5 R according to EN 197-1 [1] and the limestone filler (ρ = 2.68 g/cm^3^). The sands were both natural and siliceous. However, the medium sand from river (therefore, quite spherical, with ρ = 2.62 g/cm^3^ and water absorption of 0.40 %) while the fine sand was from dune (therefore, markedly less spherical than the medium sand, with ρ = 2.63 g/cm^3^ and water absorption of 0.20 %). A superplasticizer, third generation, polycarboxylate based was used (ρ=1.08 g/cm^3^ and solid content of 40%). The water was distilled.

All the mixes were prepared in a standard mixer for mortars [2]. The following procedure was done: all the constituent materials were previously weighed and stored in plastic containers. The powders, the sand and 80% of the total water were joined (that moment was defined as being *t* = 0) and then mixed at low speed for 120 s. Mixing was stopped for 60 s. During those 60 s de paddle was cleaned. In the last 10 s the superplasticiser and the remaining water was added. Mixing was re-started at low speed and last for 120 s. Mixing was stopped for 60 s. During those 60 s de paddle was cleaned. Mixing was re-started at hight speed and last for 30 s.

Immediately after mixing the funnel test was done and the flow time was recorded (t-funnel – see Ref. [3] for details). Then, the slump test was done and then two orthogonal distances were recorded (D-flow1, D-flow2 – see Ref. [3] for details). After that, three prismatic specimens 4 × 4 × 16 [cm] were moulded and stored in a climatic chamber at 20 °C.

At the age 23.5 h the specimens were unmoulded for being mechanically tested at the age of 24 h (with 15 min tolerance). Firstly, the tensile strength test [2] was done in the three specimens and the values recorded (TS1, TS2, TS3). From this test, two halves per specimen were obtained. Immediately after that, the six halves were tested to the compressive strength [2] (fc1.1, fc1.2, fc2.1, fc2.2, fc3.1, fc3.2).

The mix compositions contents were calculated for a batch with a volume of 1.529 L. The design of experiments was defined for a 2^5^ factorial design consisting on 32 treatment combinations augmented by 10 axial runs plus 8 central runs was used, resulting in a central composite design with a total of 50 mortar trial mix composition. Additionally, 14 extra mix compositions manually defined for required validations of response models purposes. Mix proportions were calculated based on the five independent design variables: Water_v_/Cement_v_, Superplasticyzer_m_/Powder_v_, Water_v_/Powder_v_, Sand_v_/Mortar_v_, FineSand_v_/Sand_v_. Each independent variable was evaluated at the levels -α, -1, 0, +1, +α, with α =2.37841423. The correspondence between the levels and the real values are displayed in the Table 1.

## Ethics Statement

Nothing to declare.

## CRediT Author Statement

**Lino Maia:** Investigation. all the research work.

## Declaration of Competing Interest

The authors declare that they have no known competing financial interests or personal relationships that could have appeared to influence the work reported in this paper.
